# Progressive Decline in Gray and White Matter Integrity in *de novo* Parkinson’s Disease: An Analysis of Longitudinal Parkinson Progression Markers Initiative Diffusion Tensor Imaging Data

**DOI:** 10.3389/fnagi.2018.00318

**Published:** 2018-10-08

**Authors:** Kirsten I. Taylor, Fabio Sambataro, Frank Boess, Alessandro Bertolino, Juergen Dukart

**Affiliations:** ^1^Neuroscience, Ophthalmology, and Rare Diseases, Pharma Research and Early Development, Roche Innovation Center Basel, F. Hoffmann-La Roche Ltd., Basel, Switzerland; ^2^Department of Experimental and Clinical Medical Sciences (DISM), University of Udine, Udine, Italy; ^3^Department of Basic Medical Science, Neuroscience, and Sense Organs, University of Bari, Bari, Italy; ^4^Psychiatry Unit, Bari University Hospital, Bari, Italy

**Keywords:** fractional anisotropy, mean diffusivity, Parkinson’s disease, DTI, aging

## Abstract

**Background:** Progressive neuronal loss in neurodegenerative diseases such as Parkinson’s disease (PD) is associated with progressive degeneration of associated white matter tracts as measured by diffusion tensor imaging (DTI). These findings may have diagnostic and functional implications but their value in *de novo* PD remains unknown. Here we analyzed longitudinal DTI data from Parkinson’s Progression Markers Initiative *de novo* PD patients for changes over time relative to healthy control (HC) participants.

**Methods:** Baseline and 1-year follow-up DTI MRI data from 71 PD patients and 45 HC PPMI participants were included in the analyses. Whole-brain fractional anisotropy (FA) and mean diffusivity (MD) images were compared for baseline group differences and group–by–time interactions. Baseline and 1-year changes in DTI values were correlated with changes in DTI measures and symptom severity, respectively.

**Results:** At baseline, PD patients showed significantly increased FA in brainstem, cerebellar, anterior corpus callosal, inferior frontal and inferior fronto-occipital white matter and increased MD in primary sensorimotor and supplementary motor regions. Over 1 year PD patients showed a significantly stronger decline in FA compared to HC in the optic radiation and corpus callosum and parietal, occipital, posterior temporal, posterior thalamic, and vermis gray matter. Significant increases in MD were observed in white matter of the midbrain, optic radiation and corpus callosum, while gray matter of prefrontal, insular and posterior thalamic regions. Baseline brainstem FA white matter (WM) values predicted 1-year changes in FA white matter and MD gray matter values. White but not gray matter changes in both FA and MD were significantly associated with changes in symptom severity.

**Conclusion:** Significant gray and white matter DTI alterations are observable at the time of PD diagnosis and expand in the first year of *de novo* PD to other cortical and white matter regions. This pattern of DTI changes is in line with preclinical and neuroanatomical studies suggesting that the increased spatial spread of alpha-synuclein neuropathology is the key mechanism of PD progression. Taken together, these findings suggest that DTI may serve as a sensitive biomarker of disease progression in early-stage PD.

## Introduction

Diffusion tensor imaging (DTI) of brain grey matter (GM) and white matter (WM) integrity is a potentially valuable tool to quantify progressive neurodegeneration in Parkinson’s disease (PD). In particular, two indices extracted from DTI – fractional anisotropy (FA) and mean diffusivity (MD) – are commonly applied to study tissue integrity. FA provides an anisotropy measure of water diffusion presumably reflecting preferential directions of fiber orientation whilst MD quantifies the overall diffusivity reflecting tissue density or its loss in a longitudinal setting. A key working hypothesis of the underlying pathophysiological process leading to neurodegeneration and subsequent clinical decline in PD is that alpha-synuclein pathology starting in brainstem GM and WM progressively spreads through connected white matter fiber systems to cortical GM structures during the course of the disease (Braak et al., 2003). If DTI alterations in PD indeed reflect the resulting neurodegenerative process due to alpha-synuclein pathology, these alterations would also be predicted to show a progressive spatial spread. Yet, most DTI studies to date in PD have focused on cross-sectional assessment of single regions of interest (ROI) such as substantia nigra pars compacta (SNpc) or basal ganglia. These brain regions have been shown to be affected in PD and linked with its pathognomonic motor symptoms, although heterogeneity across studies is high (see Cochrane and Ebmeier, 2013; Schwarz et al., 2013 for reviews). However, some whole-brain GM and WM studies also provided evidence for altered diffusion processes in other brain structures, including the cortex (Karagulle Kendi et al., 2008; Mole et al., 2016). Whilst MD has been consistently shown to be increased in PD, differential findings emerged with respect to the directionality of FA alterations, with studies reporting evidence of both increases and decreases in anisotropy measures (Mole et al., 2016; Atkinson-Clement et al., 2017).

Only very few studies to date focused on longitudinal characterization of DTI abnormalities in PD patients (Chan et al., 2016; Loane et al., 2016; Guttuso et al., 2018; Minett et al., 2018) and only one evaluated white matter FA and MD alterations in a *de novo* PD population (Minett et al., 2018). Additionally, none of these studies evaluated the potential GM pathology as evaluated through FA and MD. It therefore remains unclear if and to what extent FA and MD alterations represent potential early diagnostic and progression biomarkers in PD patients. Moreover, it also remains unclear if these alterations reflect symptom severity or predict disease progression in the early PD population.

Here we aimed to address the question of the value of GM and WM FA and MD as early diagnostic and progression biomarkers in a *de novo* PD population. We further evaluated the relationship between these imaging indices and clinical symptoms observed in the respective patient population.

## Materials and Methods

### Participants

The DTI sample comprised 116 Parkinson’s Progression Marker Initiative (PPMI) participants who completed 1 year follow-up: 71 with a recent diagnosis of PD and 45 healthy control (HC) participants. PD and HC groups did not differ with respect to age or gender distribution, but did differ with respect to MDS-UPDRS (Movement Disorder Society-sponsored revision of the Unified Parkinson’s Disease Rating Scale, Goetz et al., 2008) subscale and total scores, as expected (see **Table 1**). All PD patients were treatment naïve at baseline but were allowed to start PD medication upon need. Only categorical (yes/no) information was recorded on the respective treatment categories (L-dopa, dopamine agonists or other PD medication). This study was carried out in accordance with Good Clinical Practice (GCP) regulations and International Conference on Harmonization (ICH) guidelines. PPMI is a large multicenter study and each site independently received ethics approval of the protocol. All subjects gave written informed consent in accordance with the Declaration of Helsinki.

**Table 1:** Subject group characteristics.

Group	PD patients	HC	Stats (test value, df, *p*-value)
N	71	45	
Age (mean ±*SD*)	61.3 ± 9.3	59.6 ± 11	0.9,114,0.371
sex (male/female)	48/23	28/17	0.4,1,0.552
Dominant side (left/symmetric/right)	31/0/40	–	–
MDS-UPDRS tot (mean ±*SD*)	31.5 ± 12.9	2.7 ± 3.4	14.7,114,<0.001
MDS-UPDRS I (mean ±*SD*)	4.9 ± 3.3	1.9 ± 2.7	5.1,114,<0.001
MDS-UPDRS II (mean ±*SD*)	5.3 ± 4	0.1 ± 0.5	8.7,114,<0.001
MDS-UPDRS III (mean ±*SD*)	21.3 ± 8.9	0.7 ± 1.6	15.4,114,<0.001
Delta MDS-UPDRS tot over 1 year (mean ±*SD*)	2.3 ± 12.3	1.1 ± 3.2	0.6,114,0.546
Delta MDS-UPDRS I over 1 year (mean ±*SD*)	1.1 ± 3.5	0.5 ± 2.4	1,114,0.304
Delta MDS-UPDRS II over 1 year (mean ±*SD*)	1.5 ± 3.6	0.2 ± 1.2	2.3,114,0.022
Delta MDS-UPDRS III over 1 year (mean ±*SD*)	-0.3 ± 8.5	0.4 ± 1.6	-0.6,114,0.551
MoCA (mean ±*SD*)	27.3 ± 2.2	28.3 ± 1.1	-2.8,114,0.007
Delta MoCA over 1 year (mean ±*SD*, N)	-0.6 ± 2.5, 70	-1.2 ± 1.8,45	1.3,113,0.184
On L-dopa at follow-up (yes/no)	20/51	–	–
On DA at follow-up (yes/no)	26/45	–	–
On others PD med at follow-up (yes/no)	15/56	–	–
Resting tremor (yes/no)	55/16	–	–
Rigidity (yes/no/unknown)	58/13/0	–	–
Bradykinesia (yes/no/unknown)	65/6/0	–	–
Postural instability (yes/no/unknown)	2/68/1	–	–

*df, degrees of freedom; PD, Parkinson’s disease; HC, healthy controls; med, medication; SD, standard deviation; MDS-UPDRS, Movement Disorder Society-sponsored revision of the Unified Parkinson’s Disease Rating Scale; MoCA, Montreal Cognitive Assessment Test Scoring*.

### Data Acquisition

Baseline and 1-year follow-up examinations in the PPMI study included the administration of the MDS-UPDRS (Goetz et al., 2008). Cardiac-triggered DTI MR sequences were acquired on a Siemens 3T TIM Trio scanner using a 12-channel matrix head coil and a two-dimensional echo-planar DTI sequence with the following parameters: TR/TE = 900/88 ms, flip angle = 90°, voxel size = 2 × 2 × 2 mm^3^, 72 slices, 64 gradient directions with a *b*-value of 1000 s/mm^2^. One non-gradient volume (*b* = 0 s/mm^2^) was also acquired. See (Marek et al., 2011) and the online PPMI protocol^1^ for details of the PPMI study and image acquisition, respectively.

### DTI Image Processing

Details of the PPMI DTI pre-processing pipeline including non-linear distortion correction and FA and MD computation can be found on the PPMI website^2^. FA and MD maps were computed using the TEEM tool as described on the PPMI^2^. All maps were downloaded with corresponding structural T1 scans. All further pre-processing was performed using Statistical Parametric Mapping software (SPM12, Friston et al., 1994). To improve precision for longitudinal evaluation structural scans from different time points were first co-registered to a mean image from those time points for each subject. FA and MD maps were subsequently co-registered to the respective structural scans. The structural MRI was segmented into GM and WM compartments. FA and MD maps were then masked with the respective binarized compartments to obtain GM and WM FA and MD estimates and normalized into MNI space based on structural T1 information. A Gaussian smoothing kernel of 8 mm FWHM (full width at half maximum) was subsequently applied.

### Statistical Analyses

All voxel-wise statistical analyses of the imaging data were performed using SPM12. Subsequent statistical analyses of the extracted imaging eigenvariates and clinical data were computed using SPSS 25 (IBM Corp., Armonk, NY, United States). We first compared the baseline FA and MD GM and WM maps between PD and HC controlling for the effects of age and sex using two-sample *t*-tests. We then tested for group–by–time interactions (i.e., differential FA and MD trajectories across PD and HC between baseline and 1-year follow-up) using a flexible factorial design including the factors group, time and subject and controlling for sex and baseline age. To correct for multiple comparisons, an exact permutation based cluster threshold (*p* < 0.05) was applied to all voxel-wise analyses combined with an uncorrected voxel-wise threshold of *p* < 0.01 (Dukart et al., 2017). Eigenvariates adjusting for the effects of covariates of no interest were extracted from the most significant clusters identified in baseline comparisons and group–by–time interaction analyses in each contrast. Effect sizes (Cohen’s *d*) for the differentiation between PD and HC and percent changes from baseline (for longitudinal analyses only) were then computed to estimate the magnitude of observed alterations. Using these eigenvariates, we then used general linear models to evaluate whether baseline alterations in FA and MD observed in PD were predictive of current clinical severity or 1-year changes in clinical severity as measured by MDS-UPDRS total or subscale scores while controlling for age, sex and treatment mode at follow-up (as random effects, for change prediction only). Similarly, we tested if these baseline DTI alterations were predictive of 1-year FA and MD alterations observed in the PD population in group–by–time interaction analyses controlling for age and sex. Further, we evaluated using general linear models if changes observed in FA and MD over time (all entered as covariates into a single model) were related to changes in function (MDS-UPDRS I, II, III, and total scores) while controlling for the random effects of PD medication status (L-dopa, dopamine agonists or other PD medication), age and sex. Lastly, we tested if medication may have affected disease progression effects on FA and MD identified in the above group–by–time interaction analyses. For this we computed analyses of variance in PD testing for treatment–by–time interactions on FA and MD alterations identified in the longitudinal analyses, controlling for age and sex. We thereby tested for effects of all three medication types recorded in the PPMI database (L-dopa, dopamine agonists, and other PD medication).

## Results

### Imaging Alterations

**FIGURE 1:**
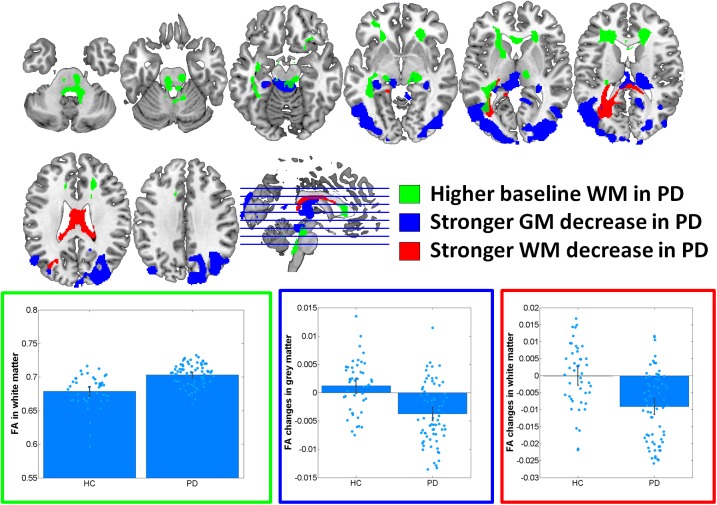
Results of voxel-based morphometry analyses of fractional anisotropy (FA) maps. Bar plots show the eigenvariates extracted from the displayed significant clusters. Outline colors of the bar plots correspond to the contrast colors of respective clusters. HC, healthy control participant; PD, Parkinson’s disease patients; GM, gray matter; WM, white matter.

**FIGURE 2:**
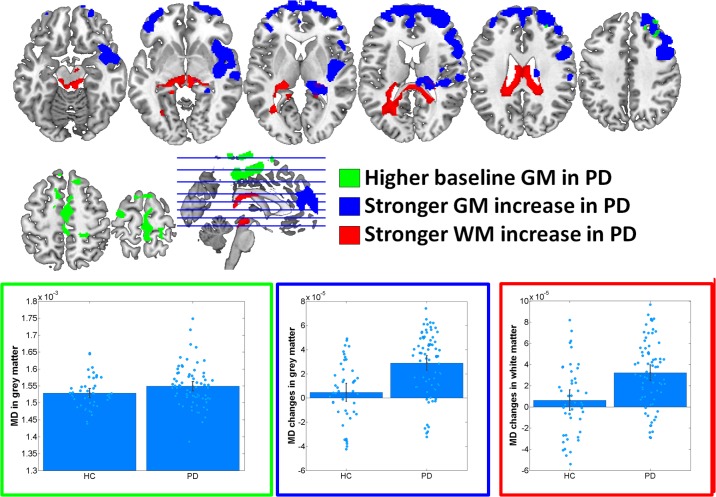
Results of voxel-based morphometry analyses of mean diffusivity (MD) maps. Bar plots show the eigenvariates extracted from the displayed significant clusters. Outline colors of the bar plots correspond to the contrast colors of respective clusters. HC, healthy control participant; PD, Parkinson’s disease patients; GM, gray matter; WM, white matter.

At baseline, significantly increased FA was observed in PD in midbrain, pons and cerebellum, anterior corona radiata, anterior corpus callosum and left inferior and inferior-occipital longitudinal fasciculus WM (Cohen’s *d* = 1.17) (**Figure 1** and **Table 2**). MD was increased in PD in bilateral primary sensorimotor and supplementary motor GM (Cohen’s *d* = 0.38) (**Figure 2**). No significant differences between PD and HC were observed in FA GM and MD WM.

**Table 2:** Results of voxel-wise analyses of FA and MD maps.

Modality	Contrast	Anatomical region	Cluster size	Exact cluster *p*-value	Peak T-value	Peak MNI coordinates
WM FA	Baseline: PD > HC	Midbrain, cortico-spinal, pontine WM and cerebellum	4118	0.012	4.61	9,-39,-35
		Anterior corpus callosum and right anterior corona radiata	2523	0.030	4.33	20,34,19
		Left inferior longitudinal and inferior-occipital fasciculus	2277	0.037	3.96	-42,-39,-8
		Left anterior corona radiata	2009	0.043	3.77	-20,17,3
GM MD	Baseline: PD > HC	Bilateral primary sensorimotor and supplementary motor areas	5486	0.047	4.3	4,-9,52
FA GM	Interaction: stronger decrease in PD	Bilateral parietal, occipital, posterior temporal, posterior thalamus, vermis	24383	0.006	5.54	-36,-81,1
FA WM	Interaction: stronger decrease in PD	Left optic radiation, anterior and middle corpus callosum	5094	0.017	5.45	4,-25,18
MD GM	Interaction: stronger increase in PD	Bilateral medial and lateral prefrontal, right insula and posterior thalamus	30012	0.038	5.02	51,10,-5
MD WM	Interaction: stronger increase in PD	Mid-brain, left optic radiation, anterior and middle corpus callosum	9260	0.015	5.11	4,-27,16

*PD, Parkinson’s disease; FA, fractional anisotropy; GM, gray matter; HC, healthy controls; MD, mean diffusivity; MNI, Montreal Neurological Institute space; WM, white matter*.

In the group–by–time interaction analyses, significant differences between PD and HC in FA changes over time were observed in both GM (Cohen’s *d* = -1.0, HC: 1.6%, PD: -4.1%) and WM (Cohen’s *d* = -0.94, HC: 0.0%, PD: -2.3%) (**Figure 1** and **Table 2**). Stronger FA GM decreases were thereby observed in PD in a cluster covering primarily bilateral parietal, occipital, posterior temporal and posterior thalamic and vermis regions. Similarly, stronger FA decreases were observed in PD in the underlying WM including the left optic radiation and anterior and middle corpus callosum. For MD, significantly stronger increases were observed in PD in GM (Cohen’s *d* = 0.95, HC: 0.5%, PD: 2.8%) and WM (Cohen’s *d* = 0.8; HC: 0.8%; PD: 3.6%) including, and bilateral medial and lateral prefrontal lobes, right insula and posterior thalamus GM and the midbrain WM, left optic radiation and anterior and middle corpus callosum (**Figure 2** and **Table 2**). These differential longitudinal FA and MD changes observed in PD were not associated with PD medication status at follow-up (all *p* > 0.85).

### Associations Between FA and MD and Function

**FIGURE 3:**
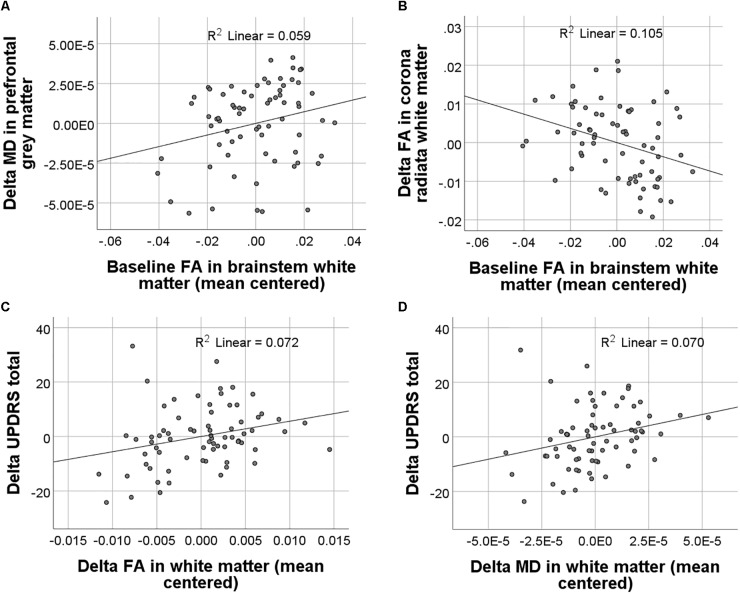
Results of regression analyses between baseline and longitudinal FA and MD values and clinical scores. Significant associations between baseline FA and longitudinal FA and MD changes are displayed in **(A)** and **(B)**. Significant associations between longitudinal FA and MD changes and changes in symptom severity as measured with the MDS-UPDRS (Movement Disorder Society-sponsored revision of the Unified Parkinson’s Disease Rating Scale) are displayed in **(C)** and **(D)**.

In PD patients, baseline FA and MD values were neither significantly associated with baseline symptom severity nor with changes in symptom severity from baseline to 1-year follow-up. Baseline FA but not MD alterations in PD significantly predicted changes in WM FA [*F*(1,65) = 7.7, *p* = 0.007] and GM MD [*F*(1,65) = 4.1, *p* = 0.048] which had been observed in the group–by–time interaction analyses described above (**Figures 3A,B**). Higher baseline FA in the brainstem predicted stronger declines in FA in the posterior corona radiata and corpus callosum. Importantly, both MD and FA WM changes were significantly associated with changes in MDS-UPDRS total scores [FA: *F*(1,61) = 4.7, *p* = 0.034; MD: *F*(1,61) = 4.6, *p* = 0.036] but not with the MDS-UPDRS subscale scores (**Figures 3C,D**). FA and MD GM changes were not significantly associated with changes in symptom severity as measured by the MDS-UPDRS.

## Discussion

We find significant baseline FA and MD increases in *de novo* diagnosed PD patients’ brainstem and subcortical WM and cortical GM. Within the first year after diagnosis, DTI abnormalities spread to significant portions of the initially unaffected cortex and WM. The progression of FA and MD changes in GM was spatially distinct, with MD changes localized to the frontal lobe regions, whereas FA changes were localized to parietal, occipital and posterior temporal regions. WM changes in both FA and MD co-localized in the posterior corpus callosum and corona radiata. Both FA and MD changes in WM but not in GM were clinically relevant, demonstrating significant correlations with changes in MDS-UPDRS total scores.

Previous research on diffusion alterations in PD have largely focused on abnormal cross-sectional findings in the substantia nigra (Cochrane and Ebmeier, 2013; Schwarz et al., 2013) or ROI (Hall et al., 2016), with a minority of studies adopting a hypothesis-free, whole-brain approach (for a review see Hall et al., 2016). With respect to anatomical location of FA and MD alterations, our cross-sectional and longitudinal findings agree well with previous studies in more advanced PD reporting FA and/or MD alterations in the midbrain (Leh et al., 2007; Cochrane and Ebmeier, 2013; Lenfeldt et al., 2013; Schwarz et al., 2013), cerebellum (Fling et al., 2013; Vercruysse et al., 2015), anterior corona radiata (Agosta et al., 2014; Lee et al., 2014), anterior corpus callosum (Melzer et al., 2012; Agosta et al., 2014; Chan et al., 2014; Lee et al., 2014; Canu et al., 2015), left inferior and inferior-occipital fasciculi (Kamagata et al., 2012; Deng et al., 2013; Lee et al., 2014) and bilateral primary sensorimotor and supplementary motor areas (Fling et al., 2013; Vercruysse et al., 2015). Most of these studies reported decreased FA and/or increased MD in the respective regions. Importantly, all of these studies focused on more advanced disease stages. In contrast, a more complex picture emerged from the present results in the *de novo* PD population with respect to the directionality of FA changes. Whilst our longitudinal data indeed suggest a faster FA decline in PD as compared to HC, at baseline, we observed only increases in WM FA in a wide-spread anatomical network including corticospinal tracts and subcortical WM. These discrepant findings with respect to FA as compared to results in more advanced PD patients point to a more complex picture of underlying pathology in particular at early clinical disease stages. Importantly, our findings are in line with two out of three previous studies in *de novo* PD reporting numerically increased FA values in white matter (Tessa et al., 2008; Zhang et al., 2016). FA directionality is often difficult to interpret in terms of underlying neuropathology. For example a selective loss of specific fiber directions in crossing-fiber regions may result in increased FA, whilst a general loss of fibers along a specific WM tract is expected to result in decreased FA. Additionally, depending on the original structure composition a neurodegenerative or a neuroinflammatory process (both being key contributors if PD) can lead to increases and decreases in FA. An increased baseline FA in *de novo* PD may therefore represent an example of the former situation or point to a different, more complex underlying neuropathology. We note that in particular the finding of increased FA in the corticospinal tract is in line with a recent *meta*-analysis reporting this region as only one showing consistently increased FA across studies (Atkinson-Clement et al., 2017). Moreover, the significant correlation observed between this increased baseline FA and longitudinal FA and MD changes suggests that higher baseline FA is indeed associated with stronger FA loss and MD increase observed in the first year of follow-up. These findings support the relevance of this baseline FA values as a potential predictor of the future spread of pathology.

Far fewer studies analyzed the longitudinal progression of DTI signals in PD. Most notably, Zhang et al. (2016) analyzed PPMI DTI data from 122 PD patients and 50 HCs using a WM and subcortical region of interest approach (ROI) based on the JHU-DTI-MNI (Type I WMPM) atlas^3^. GM DTI findings were not analyzed, and the authors selected radial and axial, as opposed to mean, diffusivity measure for their analyses. Using this ROI approach, PD patients did not differ from controls in any WM or subcortical region of interest at baseline. However, in line with the present findings, FA declined significantly more rapidly in PD patients’ substantia nigra, midbrain, thalamus, corpus callosum, and frontal white matter. FA changes in all ROIs were not associated with changes in MDS-UPDRS scores. We note that the present study’s positive relationship between FA and MDS-UPDRS changes derived from global, as opposed to ROI, FA changes. Nevertheless, the converging findings between the present and Zhang et al. (2016) studies support the use of DTI to understand WM changes in *de novo* PD. Importantly, DTI based measures may provide complementary information in addition to other imaging modalities such as volumetric and resting state functional MRI. On one side, although volumetric MRI is very sensitive to alterations in regional gray matter volume and cortical thickness it provides little insight into the underlying gray and white matter microstructural changes. Such volumetric changes presumably reflect irreversible damage due to loss of the underlying tissue. On the other side, resting state functional MRI measures provide an insight into neural dysfunction, i.e., loss of activity or connectivity (Dukart et al., 2017). However, such local functional alterations may also reflect changes in the underlying structure (i.e., due partial volume effects) or damage of remote structures, i.e., reduced input (Dukart and Bertolino, 2014). In that sense, DTI provides a complimentary insight into the underlying microstructural changes in the respective regions. As diffusion processes may be affected by any alterations in tissue organization such as inflammatory, demyelinating or other processes, DTI provides a potentially more sensitive biomarker to pick up early microstructural changes in PD prior to tissue loss detected through volumetric MRI and without confounding factors related to interpretation of resting state functional MRI.

Braak et al. (1999) demonstrated that alpha-synuclein-positive inclusions are present in the axons of PD patients, e.g., the intramedullary vagal axons from the dorsal motor vagal area, which itself contained many alpha-synuclein-positive Lewy bodies. Moreover, Iseki et al. (2001) found that ubiquitin-positive inclusions in the central nucleus of the amygdala of patient brains with dementia with Lewy bodies were also alpha-synuclein and tyrosine hydroxylase positive, suggesting that the degeneration of terminal axons of affected substantia nigra neurons were the source of the amygdala pathology in patients with dementia with Lewy bodies (Iseki et al., 2001). Consistent with this spreading model, the present study found that baseline brainstem FA WM values significantly correlated with both 1-year changes in FA WM and MD GM values. These findings indicate that DTI may serve as a valuable biomarker of both disease progression and, extrapolating from Spencer et al. (2017) preclinical study, also drug efficacy in studies of disease-modifying agents.

Burke and colleagues suggest that axonal damage in PD may occur prior to neuronal loss, as described in their “axonal dying back” model (Burke and O’Malley, 2013; Tagliaferro and Burke, 2016). The evidence for this claim derives primarily from various mouse models of PD [e.g., BAC, heterozygous null gene engrailed1 (En1), and Nurr1 transgenic mouse models], which all show axonal pathology prior to neuronal loss (for a review see Tagliaferro and Burke, 2016). Correspondingly, in humans, circa 70% of nigrostriatal dopaminergic terminals are estimated to be lost at the timepoint of the PD diagnosis, while the level of neurodegeneration has only reached 30% of substantia nigra dopaminergic neurons (Cheng et al., 2010). Moreover, a recent study of Alzheimer-related pathology suggests that DTI abnormalities may portend the downstream aggregation of pathological proteins: in a multi-model imaging study with MRI DTI and FTP tau PET, Jacobs et al. (2018) demonstrated that abnormal MD in the hippocampal cingulum bundle predicted tau accumulation in the downstream-connected posterior cingulate cortex 2 years later in amyloid positive older individuals. We note that as in synucleinopathies, intraneuronal pathologically aggregated tau proteins in Alzheimer’s disease are likewise hypothesized to spread from neuron to neuron along WM tracts (Clavaguera et al., 2009). Taken together, these findings suggest that DTI may be used not only as an early marker of alpha-synuclein pathology, but of future upstream neuronal cell loss and downstream pathological alpha-synuclein aggregation.

The present study demonstrated baseline brainstem and cortical abnormalities in *de novo* PD WM and GM with DTI, as expected, and a propagation of these abnormalities within the first year of *de novo* PD to include large swathes of cortical regions. Importantly, both the FA and MD changes were functionally relevant, as evidenced by significant correlations with changes in MDS-UPDRS total scores. The patterns of cortical WM neurodegeneration in PD mimicked those described during normal aging, with stronger FA effects in frontal and stronger MD effects in parietal regions, which together suggest a selective vulnerability of these brain regions. In the context of preclinical PD models suggesting an early affectation of axons affected by synucleinopathy (i.e., prior to neuronal loss) (Cheng et al., 2010), the present findings indicate that DTI-based measures of WM and GM integrity may represent powerful early biomarkers of disease progression in *de novo* PD.

## Data Availability Statement

The datasets analyzed for this study can be found in the PPMI data repository (http://www.ppmi-info.org/access-data-specimens/download-data/).

## Author Contributions

JD, FS, FB, and AB conceptualized the analysis plan. JD analyzed the data. KT and JD wrote the manuscript.

## Conflict of Interest Statement

The authors declare that the research was conducted in the absence of any commercial or financial relationships that could be construed as a potential conflict of interest.

## Footnotes

1 http://www.ppmi-info.org/wp-content/uploads/2018/02/PPMI-AM-13-Protocol.pdf

2 http://www.ppmi-info.org/wp-content/uploads/2011/12/DTI-processing-Pipeline3.pdf

3 http://cmrm.med.jhmi.edu
